# Economic Evaluation of a Novel Treatment of Attention-Deficit/Hyperactivity Disorder in US Motor Vehicle Drivers

**DOI:** 10.36469/001c.121305

**Published:** 2024-09-17

**Authors:** Jacie T. Cooper, John E. Schneider, Jim Potenziano, David S. Fam

**Affiliations:** 1 Avalon Health Economics, Morristown, NJ, USA; 2 Avalon Health Economics, Miami, Florida, USA; 3 Tris Pharma, Inc., South Brunswick, New Jersey, USA

**Keywords:** ADHD, driving simulation, cost impact, amphetamine, motor vehicle accidents, economic burden, young adults

## Abstract

**Background:** Attention-deficit/hyperactivity disorder (ADHD) affects approximately 4.4% of US adults. ADHD is associated with high-risk driving behavior and costly motor vehicle accidents. DYANAVEL XR (DXR) (Tris Pharma, Inc.) is a once-daily fast-acting amphetamine developed for ADHD treatment. A randomized controlled trial showed that DXR patients were 43% less likely to crash during a driving simulation than individuals taking placebo. Study outcomes suggest a DXR crash rate similar to that of a driver without ADHD, while patients treated with the current standard of care (SOC) have a 52% higher crash risk than non-ADHD drivers. **Objective:** The aim was to evaluate the economic benefits attributable to improved driving abilities and avoided crashes in DXR patients compared with patients treated with the SOC or those who are untreated. **Methods:** A cost-impact model estimated 1-year crash-related cost outcomes for DXR-treated patients compared with SOC-treated and untreated ADHD patients. SOC was assumed to consist of a combination of short-, intermediate-, and long-acting ADHD stimulant and non-stimulant medications. DXR crash risk was assumed equivalent to the non-ADHD population risk, as supported by trial data. Crash risk for untreated and SOC-treated ADHD patients were assumed to be 99% and 52% higher than the general US population, respectively. Model outcomes included the cost impact (medication- and crash-related costs) and the number of crashes, injuries, and fatalities avoided with DXR. **Results:** Treatment with DXR would avoid 0.82 crashes, 0.016 injuries, and 0.036 fatalities per year compared with untreated patients, and 0.036 crashes, 0.007 injuries, and 0.0001 fatalities per year compared with SOC-treated patients. Compared with a population of 25% SOC-treated patients and 75% untreated patients, DXR use would save an average of 4581perpersonperyearacrossallagegroupswhenpricedat80 per month, assuming all SOC-treated and untreated patients utilized DXR. When the value of quality-of-life improvement is considered, savings increase over 7-fold. **Discussion:** Outcomes suggest that DXR may be an economically beneficial treatment compared with SOC for ADHD patients. **Conclusions:** The economic model showed that DXR is cost-saving compared with no treatment and SOC by reducing the number of motor vehicle crashes in the ADHD population.

## BACKGROUND

Attention-deficit/hyperactivity disorder (ADHD) affects approximately 4.4% of adults in the United States, with an estimated 8.1% of the US population experiencing ADHD at some point in their lifetime.^1,2^ Research has shown that a relationship exists between driving performance and the presence of ADHD, as ADHD is a known risk factor for traffic accidents. ADHD is associated with risky driving behavior due to symptoms such as response inhibition, impulsivity, inattentiveness, problems controlling interference, and mood lability.^3,4^ ADHD has been correlated with increased motor vehicle accident (MVA) rates due to both distracted driving and deliberate traffic violations, resulting in a 99% increased risk of MVA for untreated ADHD patients compared with patients without ADHD.^5^ With current available medications, treated ADHD patients still have a risk of MVA 42% higher than that of patients without ADHD.^5^

MVAs can heavily impact the quality of life of those involved by causing property damage, injury, or death. Accidents are also quite expensive; in 2019 alone, MVAs cost American society $340 billion.^6^ On an individual level, the National Safety Council (NSC) reports that the economic cost of an accident can range from $5700 per vehicle for a crash with no injuries to $155 000 for a crash involving a disabling injury. Each crash causing 1 or more deaths costs an average of $1 778 000 in economic costs alone; once the comprehensive cost of lost years of life are considered, this cost skyrockets to nearly $12.5 million.^7^

Longer-acting pharmacological solutions have the potential to address these issues. DYANAVEL XR (DXR, Tris Pharma, Inc.) is a novel treatment developed for ADHD. DXR is a once-daily amphetamine tablet that is fast-acting, with treatment effects lasting throughout the day. The main difference of DXR from other formulations of extended-release amphetamine is the delivery formulation. The LiquiXR technology is capable of consistently delivering amphetamine to the patient without bursts of release or gaps in the release of the product, leading to a fast “on” and a consistent delivery for the specified time with a controlled and smooth “off.” This evolution of technology has led to a product that may better support the need and timing for individuals requiring improved focus during morning and evening driving. Evidence from a clinical trial assessing the impact of DXR on driving performance using a driving simulator showed that patients treated with DXR were 43% less likely to crash during a simulation run than patients taking placebo.^8^ DXR recipients also showed patterns of behavior associated with lower relative crash risk than placebo patients, including increased time to collision, lower breaking intensity, and shorter throttle release time when faced with a potential crash scenario. This analysis aimed to evaluate the economic benefits of using DXR to treat general ADHD, which likely stem from improved driving abilities and crashes avoided, vs no treatment and current standard of care (SOC).

## METHODS

### Model Background

This economic evaluation was informed by published literature and the outcomes from clinical trial NCT04027361, which assessed the impact of DXR on driving performance in US drivers with ADHD.^8^ To assess the economic benefits of DXR in this population, a cost-impact model was developed that captured both 1-year and lifetime economic outcomes. Crash-related costs of individuals treated with DXR were compared with those of both untreated ADHD patients and ADHD patients treated with SOC. Primary clinical endpoints for DXR and SOC were short-term and long-term driving-related treatment efficacy, defined as annual and lifetime MVA probabilities, and MVA severity categorized as property damage only, injurious, or fatal. Primary economic endpoints were expected medication costs (for DXR- or SOC-treated drivers) and expected crash-related costs. Key economic variables included the number of crashes, injuries, and fatalities avoided. Results are presented for the average adult ADHD patient and by age group.

### Patient Population

The modeled cohort was all US drivers with ADHD, as the population of interest was all patients eligible to operate a motor vehicle. According to the latest US Census (2019), there are approximately 255 200 373 adults in the United States.^9^ This is a conservative estimate of the eligible population, as young adults begin driving at 16 or 17 years of age, depending on location in the United States. The prevalence of ADHD in adults is estimated to be approximately 4.4%, equating to about 11 476 800 drivers with ADHD.^1^ According to data from the NSC, the majority of the driving population is between 25 and 64 years of age. The population distribution is shown by age group in **Table 1**.^10^

**Table 1.** Clinical and Economic Model Inputs

**Field**	**Age Group, y**								**Overall**
	**16-19**	**20-24**	**25-34**	**35-44**	**45-54**	**55-64**	**65-74**	**75+**	
Population characteristics									
Population distribution (%)	3.60	7.50	17.50	17.00	16.10	17.00	13.30	8.00	100
Medication adherence (%)	56.53	56.91	62.32	66.31	67.43	68.48	67.46	65.02	65.15
Annual crash probabilities (%)^a^									
Average US adult	23.66	24.42	12.19	9.52	8.61	6.76	4.88	3.92	10.12
Untreated ADHD	38.20	39.19	21.68	17.34	15.81	12.63	9.28	7.53	18.34
SOC-treated ADHD	33.86	34.77	18.42	14.45	13.09	10.37	7.59	6.19	15.46
DXR-treated ADHD^b^	29.98	30.78	15.77	12.15	10.95	8.61	6.31	5.18	13.16
Annual injury probabilities (%)^a^									
Average US adult	26.30	27.15	13.55	10.58	9.57	7.51	5.42	4.36	26.30
Untreated ADHD	41.58	42.63	23.81	19.09	17.42	13.94	10.26	8.33	41.58
SOC-treated ADHD	37.07	38.04	20.30	15.95	14.47	11.47	8.41	6.86	37.07
DXR-treated ADHD^b^	32.94	33.82	17.42	13.44	12.12	9.54	6.99	5.75	32.94
Annual fatality probabilities (%)^a^									
Average US adult	0.21	0.22	0.11	0.09	0.08	0.06	0.04	0.04	0.01
Untreated ADHD	0.43	0.44	0.22	0.17	0.16	0.12	0.09	0.07	0.03
SOC-treated ADHD	0.36	0.37	0.18	0.14	0.13	0.10	0.07	0.06	0.02
DXR-treated ADHD^b^	0.31	0.32	0.15	0.12	0.10	0.08	0.06	0.05	0.02
Average cost per crash ($)^a,c^									
Untreated ADHD	45 187	45 187	45 187	45 187	45 187	45 187	45 187	45 187	45 187
SOC-treated ADHD	36 124	36 035	35 128	34 442	34 244	34 032	34 129	34 552	36 124
DXR-treated ADHD^b^	27 060	26 884	25 068	23 697	23 301	22 878	23 070	23 917	27 060

^a^Probabilities for DXR assume event probabilities are equivalent to those of the average US adult.

^b^Estimates account for expected adherence impact on medication effects.

^c^Costs do not include value from QALYs.

Abbreviations: ADHD, attention-deficit/hyperactivity disorder; DXR, DYANAVEL XR; SOC, standard of care.

### Cost of Medication

For the purposes of the model, the cost of DXR to payers is estimated to be between $80 and $120 per month, based on data directly from the manufacturer, which accounts for patients paying with coupons, out of pocket, or through insurance. Assuming daily single-pill doses, this results in annual costs between $974 and $1461 per patient. Model results were generated with the cost at both ends of the range to account for uncertainty in the estimate. Untreated patients were assumed to have no ADHD-attributable medication costs.

The annual cost of SOC medication was estimated by identifying pharmacy-related costs from IBM MarketScan data reported in a published claims data analysis, which reports all costs regardless of how payments were made.^11^ Pharmacy costs were compared between drivers with ADHD and drivers without ADHD to determine excess costs due to ADHD for commercially insured, Medicaid, and uninsured patients. According to MarketScan data, 73.5% of the population utilizes commercial insurance, 13.90% utilizes Medicaid, and the remaining 12.60% are uninsured. The average annual ADHD-attributable pharmacy cost for those under commercial insurance, under Medicaid, and without insurance are $1913, $1980, and $1345, respectively. The average ADHD-related pharmacy cost was weighted by insurance type, resulting in an average annual medication cost of $1850.75 per SOC patient as of the end of Q3 of 2023, prior to the arrival of generic competition to the market.

### Medication Adherence

Adherence was measured using the IQVIA Formulary Impact Analyzer (FIA) from January 2022 to May 2023, focusing on data for all extended-release stimulants, both generic and branded. Adherence was calculated using a standard proportion of days covered (PDC) metric, which quantifies the percentage of time a patient has had access to their medication over a fixed period, defined as:

$$\begin{array}{rl} \text{PDC}=\text{ Sum of Days C} & \text{overed in Period}\\ ÷\text{ Number of Total } & \text{Days in Period} \end{array}$$

The PDC is first calculated for each patient in each cohort. The PDC is then averaged across patients to derive 1 PDC value by cohort and age group. The results are averaged again across cohorts to generate PDC values by age group alone. It was assumed that the PDC would limit both the treatment effect and cost of the medication, as patients not taking their medication would presumably take their pill the following day and thus would delay their medication costs until the 30-day supply is empty. Adherence for the overall cohort was 65%; adherence by age group is shown among the clinical model inputs in **Table 1.** These data were applied to both SOC and DXR populations.

### Annual Crash Probabilities

The probability of experiencing a crash in a given year was estimated for the general US population using 2021 crash rates from the NSC.^10^ The data were presented as the number of crashes per 100 drivers per year, which were calculated into the number of crashes per driver per year by dividing each rate by 100. These values were then converted into probabilities by assuming that this rate is constant over the 1-year period using the equation *p* = 1− exp(−*rt*), where *r* is the rate of crashing, and *t* is the period of interest (1 year). The annual probabilities of crashing in 1 year for the average US adult are presented by age group in **Table 1**. These data generally show that younger drivers are much more likely to crash than older drivers.

To generate an average probability of crashing for the entire cohort, the average crash rate for each age group was weighted by the percentage of the population in each respective age range (**Table 1**). This resulted in an average rate of crashing of 0.11 and an annual probability of crashing of 10.12%.

### Lifetime Number of Crashes

The number of crashes over a lifetime for the average US driver was estimated using the average rate of crashes per year from the annual crash probability calculation (0.11). This rate was applied to the average number of years an individual in the United States spends driving to generate the average number of crashes over a typical lifetime. The average number of driving years for the US adult was calculated as 67, assuming a driving start age of 16 and a driving stop age of 83 years.^12^ A rate of 0.11 crashes per year for 67 years generated an estimated 7.15 crashes per person over a lifetime. Although the generally accepted range for number of crashes in a lifetime is 3 to 4, this number is likely underestimated as many accidents are not reported to the police.^13^ Alternatively, the percentage of licensed drivers estimated to have been involved in a reported or unreported crash in 2019 is much higher at 9.7%, equating to an annual rate of 0.105, which is very close to the model’s estimated 10.12% probability and 0.11 annual rate.^13^

### DXR Impact on Crash Probabilities

Results from a clinical trial driving study (NCT04027361) assessing the impact of DXR treatment on driving abilities (compared with untreated ADHD patients) were utilized to estimate the impact of DXR on crash probabilities in the economic model. The study consisted of 3 ADHD patients’ simulated drives for each participant, 1 each at baseline (before treatment), 45 minutes after receiving treatment, and 10 hours after receiving treatment. The study measured risk of crashing by capturing time to collision (TTC), which was defined as “a moment-to-moment measure (in seconds) of how close in time the participant’s vehicle came to colliding with a lead vehicle. This value is a function of the distance between the 2 vehicles and their relative speeds (velocities).”^10^ Higher TTC values represent a lower risk of collision, while lower TTC values represent a higher risk of collision.

Median TTC values from the simulated baseline drive and the simulated drive at the 45-minute and 10-hour mark were compared to estimate the influence of DXR on crash probability, which was quantified as the baseline-adjusted percent difference in TTC change for DXR patients vs placebo patients. Driving outcomes were substantially improved at both the 45-minute and 10-hour timepoints. The untreated patient population had a median TTC of 5.52 seconds at baseline and 4.67 seconds at 10 hours, resulting in a TTC decrease of 0.85 seconds compared with baseline. DXR patients had a median TTC of 4.71 seconds at baseline and 8.36 seconds at 10 hours, resulting in a 3.65-second improvement. At the 45-minute timepoint, patients experienced a median TTC decrease of 0.40 seconds from baseline with placebo and a median TTC increase of 1.97 seconds with DXR. Comparing these time differences resulted in a 529% difference in TTC for DXR vs placebo at the 10-hour timepoint and a 593% difference at the 45-minute timepoint. The model utilized results from the 10-hour timepoint, as this emphasizes the longevity of the medication effect.

If a 529% decrease in TTC from the 10-hour time point were applied to an annual probability of crashing (any value between 0% and 100%), an unrealistic crash probability would be generated for DXR patients. Thus, the model assumed that the crash probability or rate for ADHD patients utilizing DXR was equivalent to the crash probability or rate of the general US population. This was a cautious estimate, as the data suggest that DXR may improve driving abilities for ADHD patients compared with the average US adult. This is based on 2 factors. First, the treatment could feasibly increase awareness and focus of the driver to the point where it exceeds that of the average individual without ADHD. Although no direct comparative data exist between ADHD patients using amphetamines and non-ADHD individuals, there is evidence that use of amphetamines can increase reaction time and physical performance.^14,15^ Second, the probability of collision for the average US adult was based on a cohort without ADHD patients excluded, which may leave opportunity for improvement upon that average.

### Crash Probabilities for Drivers with ADHD

The probability of crashing for untreated, SOC-treated, and DXR-treated ADHD patients was estimated using odds ratios (ORs) from published literature. In a US cohort study exploring the association between ADHD medication use and MVA risk, Chang et al identified an OR of 1.99 for crashes for untreated ADHD patients compared with non-ADHD control subjects, and an OR of 1.42 for ADHD patients using medication compared with non-ADHD control subjects.^5^ This implies that untreated ADHD patients have a 99% higher risk of crashing than non-ADHD patients, and medicated ADHD patients have a 42% higher risk of crashing than non-ADHD patients. As it was previously assumed that the risk of crash for a driver treated with DXR would be equivalent to the risk for a driver without ADHD, the model assumed that DXR patients have an OR of 1.00.

To apply these ORs to current crash probabilities, the probabilities were first transformed into odds. The ORs were then applied multiplicatively to the odds, which were then transformed back into annual probabilities. PDC adherence was also applied to both treatment arms. The resulting probabilities of crashing each year were calculated by treatment type for each age group (**Table 1**).

The number of crashes over a lifetime was calculated using the overall crash probabilities as shown in **Table 1**. These probabilities were converted into rates using the formula *r* = −[ln (1 − *P*)] / *t*, resulting in rates 0.20, 0.17, and 0.14 for the untreated, SOC-treated, and DXR-treated ADHD patients, respectively. Multiplying these rates by an average of 67 driving years gives 13.58, 11.25, and 9.45 crashes over a lifetime for untreated, SOC-treated, and DXR-treated patients, respectively.

### Probability of Injury or Fatality

To estimate the probability of crash-related injury and fatality, data from a report by the National Highway Traffic Safety Association (NHTSA) was utilized, which quantified the average cost of an MVA.^6^ These data showed that of all crashes, 76.8% are property damage only, 23.0% cause at least 1 injury, and 0.2% cause at least 1 fatality. In the ADHD population, it is expected that the distribution will be more heavily skewed towards “severe” (injury and fatality) crashes due to riskier driving behaviors.^16,17^ According to a study on driving habits of young adults with ADHD, the probability of a severe crash is much higher for ADHD patients than control patients (60% vs 17%).^18^ To correct the crash-type distribution for this effect, the serious crash percentage for the control population was adjusted to match the NHTSA-reported severe crash probability in our general US population by increasing the value by 36.8%. The same adjustment for the ADHD population led to an estimated 82.1% of crashes containing either an injury or a fatality. The NHTSA-reported data showed that of the severe crashes, 98.98% of them contain an injury, and 1.02% contain a fatality. It was therefore concluded for the ADHD population that 17.9% of MVAs are property damage only, 81.2% cause an injury, and 0.8% cause a fatality.

NHTSA data showed that each injury crash contains an average of 1.37 injuries, and each fatality crash comprises an average of 1.09 fatalities. Applying these values multiplicatively along with the crash probabilities shown in **Table 1** and the distribution of crash severities described above enabled the calculation of probability estimates for crash-related injury and fatality. The resulting crash and fatality probabilities for each age group are displayed in **Table 1**.

### Cost of an MVA

The average cost of a crash was calculated using evidence from the 2023 NHTSA report by Blincoe et al.^6^ Cost fields considered in that analysis were medical care, emergency medical services, market productivity, household productivity, insurance administration, workplace costs, legal costs, congestion, and property damage. The study also quantified the cost of lost quality of life using quality-adjusted life-years (QALYs). Further detail on all cost fields is presented in the **Online Supplementary Material**.

Considering all cost components, the average cost of a crash resulting in property damage only is $9297, the cost per injury crash is $261 189, and the cost per fatal crash is $12 222 571. When weighted by the frequency of each type of crash, the average cost per crash for an untreated driver with ADHD is $315 994. The majority of this crash cost is driven by the quantification of QALYs, which accounts for 85.7% of costs. Since the base-case analysis only considered tangible costs, the QALY value was removed, making the average cost per crash $45 187 for the untreated ADHD population.

Crash severity positively correlated with the presence of ADHD symptoms, resulting in more injury- and fatality-causing crashes in the ADHD population compared with the general population.^16,17^ The average cost per crash for the average driver was $173 124, compared with $315 994 for an untreated ADHD patient. It was assumed that patients treated with DXR would have driving abilities comparable to that of the general population and patients crashing while those treated with DXR would have an average crash severity correlating with a cost of $173 124. As the treatment effect of SOC was expected to be inferior to that of DXR, the crash cost for patients treated with SOC was assumed to be the midpoint between untreated and DXR-treated crash costs, costing $244 559 per crash. Without the economic value of QALYs, each crash would cost approximately $24 757 when treated with DXR and $34 972 when treated with SOC. The cost per crash for each arm by age group is shown in **Table 1**, accounting for medication adherence and without QALY value.

## RESULTS

Model results are presented as 1-year and lifetime estimates of total cost saved and avoided crashes, injuries, and fatalities. One-year results are presented by age group, while lifetime results are presented for the average cohort. Outcomes are shown for DXR costs at both $80 and $120 per 30-day supply to account for the uncertainty in cost.

### One-Year Outcomes

Clinical outcomes for a 1-year timeframe are shown in **Table 2**. In a 1-year time frame, treatment with DXR would avoid an average of 0.05 crashes, 0.01 injuries, and 0.0001 fatalities per patient compared with untreated patients, and 0.02 crashes, 0.001 injuries, and 0.00001 fatalities per patient compared with SOC-treated patients across all age groups. When considering the entire US adult ADHD cohort estimated at 11 476 816 patients, DXR would avoid 512 391 crashes, 91 281 injuries, and 776 fatalities if utilized by every US driver, assuming that 75% of the ADHD population is currently untreated and 25% is treated with SOC.

**Table 2.** One-year Clinical Outcomes by Age Group and Treatment Arm (per Patient)

**Age Group, y**		**Untreated**			**SOC-Treated**			**DXR-Treated**	
	**Crashes**	**Injuries**	**Fatalities**	**Crashes**	**Injuries**	**Fatalities**	**Crashes**	**Injuries**	**Fatalities**
16-19	0.38	0.42	0.004	0.34	0.37	0.004	0.30	0.33	0.003
20-24	0.39	0.43	0.004	0.35	0.38	0.004	0.31	0.34	0.003
25-34	0.22	0.24	0.002	0.18	0.20	0.002	0.16	0.17	0.002
35-44	0.17	0.19	0.002	0.14	0.16	0.001	0.12	0.13	0.001
45-54	0.16	0.17	0.002	0.13	0.14	0.001	0.11	0.12	0.001
55-64	0.13	0.14	0.001	0.10	0.11	0.001	0.09	0.10	0.001
65-74	0.09	0.10	0.001	0.08	0.08	0.001	0.06	0.07	0.001
75+	0.08	0.08	0.001	0.06	0.07	0.001	0.05	0.06	0.000

Abbreviations: DXR, DYANAVEL XR; SOC, standard of care.

**Figure 1.**
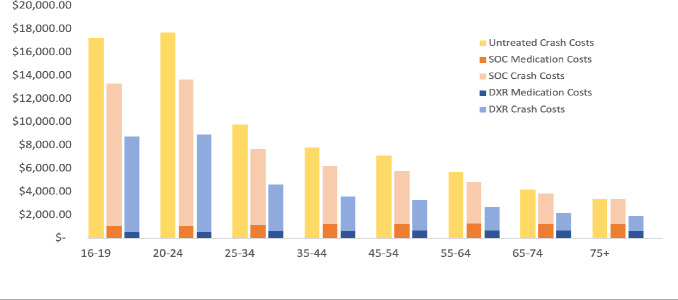
One-Year Economic Outcomes by Age Group and Treatment Arm Abbreviations: DXR, DYANAVEL XR; SOC, standard of care.

Per-patient costs are displayed by cost driver, age group, and treatment arm in **Figure 1**. DXR would save an average of $4337.83 per patient compared with untreated patients and $2669.86 per patient compared with SOC-treated patients in a 1-year time frame when priced at $80 per 30-day supply. At $120 per 30-day supply, DXR would save $4030.54 and $2362.56 per-patient compared with untreated and SOC-treated patients, respectively. Per-patient cost savings by age group and treatment arm are shown in **Table 3**. At the cohort level, DXR would save over $44 million when priced at $80 per 30-day supply and over $41 million when priced at $120 per 30-day supply across all age groups, assuming 75% of the comparator is untreated and 25% are treated with SOC.

**Table 3.** One-Year Cost Savings by Age Group and Treatment Arm (per Patient)

**Age Group, y**	**DXR 30-Day** **Cost ($)**	**Vs Untreated ($)**			**Vs SOC-Treated ($)**			**Vs Weighted Population ($)**		
		ADHD	Crashes	Total	ADHD	Crashes	Total	ADHD	Crashes	Total
16-19	80	-551	9040	8489	496	4073	4569	-289	7798	7509
	120	-826	9040	8214	220	4073	4294	-564	7798	7234
20-24	80	-554	9303	8749	499	4197	4696	-291	8027	7736
	120	-832	9303	8472	222	4197	4419	-568	8027	7459
25-34	80	-607	5761	5154	546	2483	3029	-319	4941	4623
	120	-910	5761	4850	243	2483	2726	-622	4941	4319
35-44	80	-646	4875	4229	581	2064	2645	-339	4172	3833
	120	-969	4875	3907	258	2064	2322	-662	4172	3511
45-54	80	-657	4516	3859	591	1901	2492	-345	3862	3517
	120	-985	4516	3531	263	1901	2164	-673	3862	3189
55-64	80	-667	3670	3003	600	1531	2131	-350	3135	2785
	120	-1000	3670	2670	267	1531	1798	-684	3135	2452
65-74	80	-657	2678	2021	591	1112	1704	-345	2287	192
	120	-986	2678	1693	263	1112	1375	-673	2287	1613
75+	80	-633	2118	1485	570	882	1452	-332	1809	1160
	120	-950	2118	1168	253	882	1135	-649	1809	1476

Abbreviations: ADHD, attention-deficit/hyperactivity disorder; DXR, DYANAVEL XR; SOC, standard of care.

### Lifetime Outcomes

Over a lifetime, DXR use would avoid 4.12 crashes, 1.30 injuries, and 0.01 fatalities per person compared with untreated ADHD patients, and 1.80 crashes, 0.57 injuries, and 0.005 fatalities per person compared with SOC-treated ADHD patients over a lifetime. At the cohort level, DXR use would avoid over 47 million crashes compared with untreated patients and 20 million crashes compared with SOC-treated patients. Graphical depiction of lifetime clinical outcomes can be found in **Table S1**.

When priced at $80 per 30-day supply, DXR is expected to save $332 660 over a lifetime compared with an untreated patient and $194 278 compared with a SOC-treated patient. At $120 per 30-day supply, savings decrease to $312 071 compared with an untreated patient and $173 689 compared with a SOC-treated patient. Assuming a comparator mix of 75% untreated patients and 25% SOC-treated patients, DXR would save an average of $298 064 and $277 475 per-patient when priced at $80 and $120 per 30-day supply, respectively. Lifetime economic outcomes by treatment arm are displayed in **Figure S1**.

### Deterministic Sensitivity Analysis

**Figure 2.**
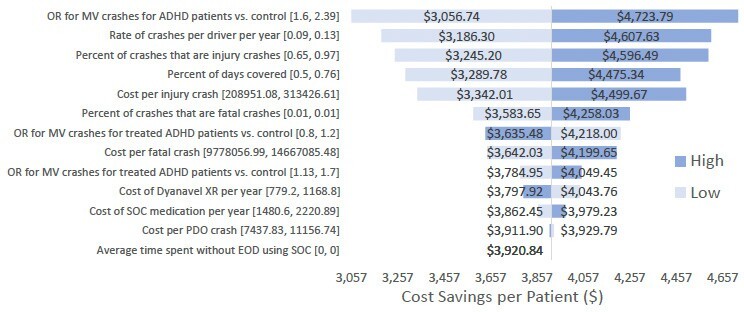
One-way Deterministic Sensitivity Analysis Tornado Diagram Abbreviations: ADHD, attention-deficit/hyperactivity disorder; DXR, DYANAVEL XR; SOC, standard of care.

A one-way deterministic sensitivity analysis was conducted on all major parameters of the base-case model to assess the robustness of potential economic savings from DXR. The analysis varied each input independently by 20% in either direction and recorded the change in savings to the 1-year per-patient results, assuming a comparator population containing 75% untreated and 25% SOC-treated ADHD patients. **Figure 2** shows the resulting tornado diagram, displaying the variable that the model was most sensitive to at the top of the graph, and the variable the model was least sensitive to at the bottom of the graph. Dark blue bars represent a 20% increase in the input value, while light blue bars represent a 20% decrease in the input value.

The vertical axis of the graph represents the cost savings based on the model’s base case, using an $80 30-day cost of DXR treatment and excluding value from extending end-of-day coverage and QALY value. Analysis results show that the model remains robust to reasonable variation in input parameters, with all analyses generating positive cost savings for DXR. The model is most sensitive to changes in the OR for untreated patients compared with non-ADHD patients, the rate of crashes per year, and the percentage of crashes that cause injury. As the base case does not consider end-of-day coverage, the model does not react to changes in this value.

### Scenario Analyses

**Scenario 1: End-of-day coverage:** The lengths of effect for ADHD medications vary substantially. Long-acting drug effects last for a maximum of 16 hours, medium-acting drugs last for about 8 hours, and short-acting drugs last for about 4 hours.^19^ Given the variation in lengths of medication effects, there is a considerable portion of time each day that SOC patients may be left “untreated,” which can impact treatment effects and therefore have cost implications. Assuming the average person spends 16 hours of the day awake and patients using short-acting drugs take 2 pills per day, patients using medium-acting or short-acting drugs spend about 8 hours each day without end-of-day coverage. According to published data from Verispan’s Vector One National, 49% of ADHD patients utilize long-acting medications, 5% use medium-acting medications, and 46% use short-acting medications.^20^ Given this distribution, the average SOC patient spends 25.5% of their day unprotected, without coverage by their medication’s treatment effect.

This was incorporated into the model as a scenario analysis by assuming that ADHD patients utilizing SOC receive the crash probabilities of untreated ADHD patients 25.5% of the time. For the remaining 74.5% of the model, they received a lowered probability of crashing from the SOC medication treatment effect. As a long-acting medication, DXR is assumed to maintain clinical effectiveness for 16 hours, represented in the model by 100% end-of-day coverage. This is supported by clinical data, where the crash probabilities for DXR remain below baseline after 16 hours.

Considering end-of-day coverage impacts to the SOC-treated population increases the 1-year crashes per patient from an average of 0.155 to 0.162 per patient. In turn, the impact from DXR on crashes, injuries, and fatality rates are increased from 0.045, 0.008, and 0.0001, respectively, to 0.046, 0.009, and 0.0001, respectively. Economically, this translates to an approximated $65 per patient.

**Scenario 2: Quality-of-life value**: The economic value of quality of life was incorporated as described in the analysis of the economic impact of MVAs by Blincoe et al.^6^ In this report, the QALY is utilized to capture the value of injuries or fatalities. The QALY is a measure ranging from 0 to 1, where 1 represents a year of perfect health and 0 represents death. The societal cost of QALY losses vary by injury severity and range from $0 for a crash with vehicle damage only to $9 651 851 for a crash resulting in a fatality. When QALY value is considered, it represents 85.7% of the total cost of crashing. The cost per crash increases to $315 994, $244 559, and $173 124 for the untreated, SOC-treated, and DXR-treated populations, respectively. This results in a substantial increase in potential savings from DXR when priced at either an $80 or $120 30-day supply to nearly $34 000 per patient per year compared with an untreated patient and over $15 000 per patient per year compared with a patient treated with SOC.

## DISCUSSION

This analysis analyzed the benefits of utilization of DXR for drivers with ADHD compared with drivers with ADHD who are untreated or utilizing SOC treatment. Model outcomes were driven by reduced crash probabilities resulting in fewer crash-related injuries, deaths, and economic costs. Results show clinical benefit when treated with DXR compared with no treatment or SOC treatment options, avoiding on average 0.05 crashes, 0.01 injuries, and 0.0001 fatalities per patient per year compared with untreated patients, and 0.02 crashes, 0.001 injuries, and 0.00001 fatalities per patient per year compared with SOC-treated patients across all age groups. Patients are expected to save a minimum of $3613 per patient per year using DXR, and these savings increase more than 8-fold when the economic value of quality of life is considered.

The results of this model illuminate the importance of effective ADHD medications by quantifying the number of MVAs that could be avoided with appropriate treatment of ADHD symptoms. The model shows that DXR could avoid over 48 million crashes compared with untreated patients and 21 million crashes compared with SOC-treated patients. The analysis also reveals the potential driving-related economic benefit of treating ADHD with DXR, as potential societal savings reach over $340 trillion per year.

This study has several limitations. First, the economic analysis relies on performance data from a driving simulation study that utilized placebo as the comparator. Thus, study evidence does not directly reflect improved driving abilities for drivers utilizing DXR compared with the SOC treatment that is analyzed in the analysis. Additionally, the general population of US drivers is utilized as a proxy for drivers without ADHD for model computation. As a result, the impact of ADHD on crash statistics may have been underestimated, as data from patients with ADHD was included in driving performance estimates of the general population. Finally, ADHD prevalence was estimated in literature to be 4.4%. This value reflected patients between the ages of 18 to 44, while our analysis assesses patients up to 83 years of age. The total US ADHD cohort costs presented in the model may therefore be slightly overestimated, as driving prevalence tends to decrease with age.

This analysis considered only the clinical and economic impact of DXR’s on driving abilities in an ADHD population. However, the findings suggest that there could be significant value in other types of outcomes, including general care costs, substance abuse, traffic violations (without crashing), and general quality of life.^21^ Further analyses to investigate the clinical and economic potential of DXR in these areas are needed.

## CONCLUSIONS

Model results show that DXR is cost-saving when priced at both $80 and $120 per month compared with untreated and SOC-treated patients when considering reductions in MVAs in the ADHD population. Savings are driven by reductions in crash rates and lower severity of crashes when patients are utilizing treatment. When the societal economic value of quality of life is considered, per-patient annual savings increase to over $34 000 compared with untreated ADHD patients and $15 000 compared with SOC-treated patients.

## Supplementary Material

Online Supplementary Material
