# Supplementary material for: Economic Evaluation of a Novel Treatment of Attention-Deficit/Hyperactivity Disorder in US Motor Vehicle Drivers
Source: J Health Econ Outcomes Res. 2024 Sep 17;11(2):66–73. doi: 10.36469/001c.121305 (PMC11413859; doi:10.36469/001c.121305)
Supplement: Online Supplementary Material [file jheor_2024_11_2_121305_246164.pdf]

Journal of Health Economics and Outcomes Research

Online Supplementary Material

Economic Evaluation of a Novel Treatment of Attention Deficit Hyperactivity Disorder in US Motor Vehicle Drivers. *JHEOR*. 2024;11(2):66-73. doi:10.36469/jheor.2024.121305

Cost Component Descriptions as Reported by Blincoe et al[6]

Figure S1: Lifetime Clinical Outcomes (per Patient)

Figure S2: Lifetime Economic Outcomes (per Patient)

This supplementary material has been provided by the authors to give readers additional information about their work.

This is an open-access article distributed under the terms of the Creative Commons Attribution 4.0 International License (CCBY-4.0). View this license's legal deed at http://creativecommons.org/licenses/by/4.0 and legal code at http://creativecommons.org/licenses/by/4.0/legalcode for more information.

Cost Component Descriptions as Reported by Blincoe et al[6]

Human capital costs: Tangible losses that result from motor vehicle crashes. They define the value of resources that are used or that would be required to restore crash victims, to the extent possible, to their pre-crash physical and financial status. These are resources have been diverted from other more productive uses to merely maintain the status quo. These costs, which can be estimated through empirical measurements, include medical care, lost productivity, legal and court costs, insurance administrative costs, workplace costs, congestion impacts (travel delay, excess fuel consumption and pollution), and property damage.

Medical care: The cost of all medical treatment associated with motor vehicle accident injuries including that given during ambulance transport. Medical costs include emergency room and inpatient costs, follow-up visits, physical therapy, rehabilitation, prescriptions, prosthetic devices, and home modifications.

EMS: Police and fire department response costs.

Market productivity: The present discounted value (using a 3% discount rate) of the lost wages and benefits over the victim's remaining life span.

Household productivity: The present value of lost productive household activity, valued at the market price for hiring a person to accomplish the same tasks.

Insurance administration: The administrative costs associated with processing insurance claims resulting from motor vehicle crashes and defense attorney costs.

Workplace costs: The costs of workplace disruption that is due to the loss or absence of an employee. This includes the cost of retraining new employees, overtime required to accomplish work of the injured employee, and the administrative costs of processing personnel changes.

Legal costs: The legal fees and court costs associated with civil litigation resulting from traffic crashes.

Congestion costs: The value of travel time delay for people who are not involved in traffic crashes but who are delayed in the resulting traffic congestion from these crashes, as well as the value of excess fuel consumed, greenhouse gases, and criteria pollutants emitted due to traffic congestion caused by the crash.

Property damage: The value of vehicles, cargo, roadway features, and other items damaged in traffic crashes.

Value of a statistical life: A measure of consumer's implied willingness to pay to avoid the risk of death. Estimated at $10.9 million.

Quality of life: The metric commonly used to value these nonfatal injury losses is the quality adjusted life year or QALY, a health outcome measure that assigns a value of 1 to a year of perfect health and a value of 0 to death. QALY loss is determined by the duration and severity of the health problem, with a full year of QALY loss being equivalent to the loss of a full year of life in perfect health.

Figure S1. Lifetime Clinical Outcomes (per Patient)

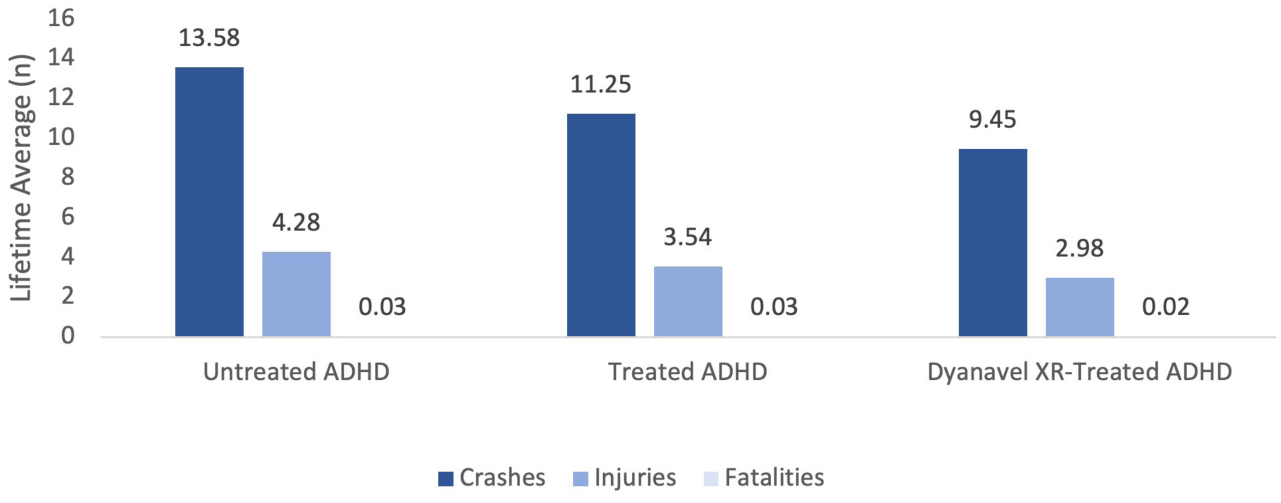

Abbreviation: ADHD, attention-deficit/hyperactivity disorder.

Figure S2. Lifetime Economic Outcomes (per Patient)

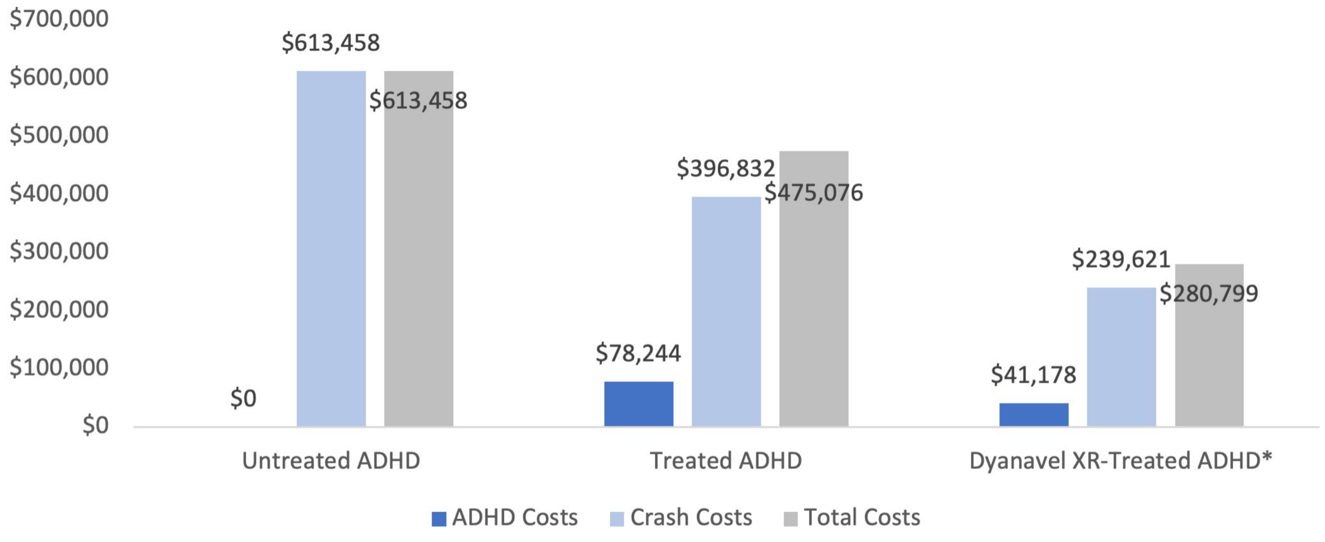

Abbreviation: ADHD, attention-deficit/hyperactivity disorder..
